# Modeling earthquake-induced wavefields and stresses in alpine mountains with extreme topography

**DOI:** 10.1038/s41598-025-08218-5

**Published:** 2025-07-04

**Authors:** Fabian Limberger, Georg Rümpker, Jan Philipp Kruse, Thibault Duretz

**Affiliations:** 1 Institute of Geosciences, Goethe-University Frankfurt, 60438 Frankfurt am Main, Germany; 2 Frankfurt Institute for Advanced Studies, 60438 Frankfurt am Main, Germany

**Keywords:** Geophysics, Seismology, Natural hazards

## Abstract

Earthquakes can trigger slope instabilities such as rockfalls, landslides, and avalanches, posing a significant hazard for residents and infrastructures, particularly in mountainous regions. This risk is further exacerbated by global warming and permafrost degradation, which destabilize surfaces. Hence, our study investigates earthquake-induced wave dynamics at mountain summits, particularly at the Matterhorn (Switzerland) and Tre Cime di Lavaredo (Italy). The selected sites represent exceptional cases of isolated peaks. While these landforms are rare even within alpine environments, they offer crucial boundary cases to explore the upper limits of topographic amplification under seismic excitation. Full wavefield modeling is utilized to simulate the induced resonant oscillations and amplification of seismic signals at the summits compared to adjacent valleys. The simulated amplification (up to 10 times) in the summit depends on the characteristics of motion direction, topography, and presence of permafrost. Major resonance modes are identified at Matterhorn at frequencies of 0.4 Hz and 1.4 Hz. Higher resonance frequencies above 2 Hz are obtained at the smaller rock formation Tre Cime di Lavaredo, indicating mountain-specific resonances. We demonstrate that the presence of a permafrost body inside the mountain tends to reduce seismic amplification by up to 30%. However, this effect is dependent on the amount of permafrost and the wavelength of the seismic waves. Locations of potential slope instabilities on the mountain’s surface are identified based on the dynamic stress changes during the simulated earthquake. We find that locations of stress amplification are mainly at the mountain flanks and are influenced by azimuthal characteristics of the incoming wave. The approach and findings presented in our study have the potential to improve hazard assessments for earthquake-induced slope instabilities, focusing on mountains with extreme geometries.

**Supplementary Information:**

The online version contains supplementary material available at 10.1038/s41598-025-08218-5.

## Introduction

Landslides and rockfalls are significant hazards for residents and infrastructures, especially in mountainous regions. Their occurrence is increasing due to climate warming and the subsequent degradation of permafrost^1–3^, as well as heavy rainfall^4–8^. Moreover, seismic activity and waves from earthquakes can induce landslides and rockfalls, or destabilize mountain slopes progressively. This is a phenomenon that is recorded globally^9–11^ and observed on regional scales, e.g., Italy^12^, Japan^13^, New Zealand^14^, China^15^, Guatemala^16^, Alaska^17^, or Papua New Guinea^18^. Furthermore, earthquakes have the potential to induce snow avalanches^19^.

Seismic wave amplification in mountainous regions, particularly during earthquakes, is a known phenomenon^20–22^. Previous experimental and numerical studies have investigated site effects and seismic amplification due to topography. Weber et al.^23^ conducted ambient noise measurements at the summit of Matterhorn and observed significant signal amplification of up to 9 times on average at the mountain summit compared to nearby valleys. Furthermore, they suggest the high potential for earthquake-induced rockfalls or landslides due to the measured and simulated resonances at distinct frequencies. Massa et al.^24^ and Weber et al.^23^ also showed the existence of preferential motion directions within the topographic formation, indicating complex wavefield dynamics resulting in resonances and signal modulations in the mountains. The importance of considering full wave form modeling to analyze and assess earthquake-induced landslides is shown by Dahal et al.^25^.

Previous numerical studies have mainly utilized generic two-dimensional models to estimate site amplifications considering factors such as wave frequencies, material properties, internal structures, and topographic features^26–30,65^. These studies have shown that topographic features can significantly enhance seismic signal amplification, particularly with steeper slopes and strong material contrasts. Additionally, the incidence angle of seismic waves influences amplifications^31^. Moreover, Gischig et al.^66,67^ extensively investigated the seismic response of deep-seated rock slope instabilities using numerical modeling based on 2D generic models to understand how seismic waves interact with unstable slopes and contribute to landslide hazards​. Their study explored different amplification phenomena, including topographic effects, material contrasts, and the influence of internal fracturing, to assess their role in enhancing co-seismic slope deformation​. Additionally, they examined how repeated seismic loading over time induces fatigue in rock masses, progressively weakening slopes and increasing their susceptibility to catastrophic failure​. Contrarily to site amplification, the presence of glaciers in the valley or frozen layers in the subsurface have the potential to damp and reflect waves and hence to reduce site amplifications^29,32^. To model resonance frequencies, Weber et al.^23^ performed numerical modal analyses, while wavefield dynamics were neglected. The development of seismic resonances in complex structural environments, such as volcanoes, was studied, e.g., by Jousset et al.^33^, Sturton and Neuberg^34^, and Limberger and Rümpker^35^. However, the complex seismic response of specific mountains is poorly modeled.

Potential damping effects of subsurface permafrost layers on wave amplifications and elastic properties of the rock were studied using experimental and numerical methods^32,36,37^. Lindner et al.^38^ analyzed seismic long-term measurements from the mountain Zugspitze (Germany) and found that seasonal permafrost melts affect seismic wave travel times and hence velocities. Nevertheless, the potential influence of permafrost degradation inside mountains on seismic amplification at summits has not been adequately explored or modeled in existing research, despite its relevance in the context of climate change promoting mountain instabilities.

The emission of seismic waves through rock masses causes a dynamic stress perturbation that adds up to the background static stress fields^39^. The effect of dynamic stress perturbations on seismic triggering has been studied based on both theory and observations^40,41^. While their effect on aftershock sequences is debated^42–45^, they are often involved to explain seismic sequences occurring in volcanic and fluid-rich environments^45–48^. In the context of landslides and rockfalls, the role of dynamic stress has been addressed in models relying on modal analysis^49^ and mechanical analysis^39,50^. The effects of fractured structures inside rocks on slope instabilities were studied by Burjánek et al.^51^ and Kleinbrod et al.^64^ using observational data linked with numerical simulation of ambient vibrations. However, these studies do not specifically consider temporal earthquake-induced wavefield and stress dynamics in realistic high-altitude alpine environments including permafrost effects and full-waveform modeling.

In our study, we address these gaps by utilizing highly resolved digital elevation models (DEM) and numerical models to estimate the characteristics and spatial distribution of frequency-dependent signal amplifications at prominent topographic structures. While topographic amplification factors of mountains with common shapes are studied in numerous publications, we model the Matterhorn in Switzerland and Tre Cime di Lavaredo (Dolomites) in Italy as case of two extreme examples in two regions that are known for frequent major rockfalls. Nevertheless, the two mountains are characterized by considerably different sizes. Through full-wavefield simulations, we investigate how these exceptional mountains respond to incoming seismic waves. Additionally, we examine the potential impact of permafrost degradation on seismic signal amplification at the Matterhorn’s summit. Finally, we establish a linkage between the simulated peak ground velocities at the mountain and stress field dynamics, offering insights into predictions of location-dependent slope instabilities during earthquakes. Unlike the existing studies mentioned, we focus on topographic effects on incoming waves from earthquakes, which might act as a special boundary condition for triggering slope instabilities. Hence, we use simplified models excluding actual slope instabilities and internal structures such as faults, layers, and fractures, which might have further effects in addition to topographic controls. We deliberately selected two highly unusual mountain geometries, both characterized by isolated, steep, and slender structures. Although such formations are not representative of the majority of alpine or global mountain landscapes, they serve as critical boundary cases to investigate the upper limits of seismic signal amplification caused by topography. Hence, the rarity of such extreme geomorphologies implies that the results from our numerical study can be hardly generalized. Instead, this motivates the investigation of the seismic behaviour of mountains at the most amplification-prone end of the spectrum, not considered by most existing studies.

## Model configuration and methodology

### Software

The wavefield modeling code used in this study is part of the Salvus software package^53^, which solves the elastodynamic wave equation using spectral elements. Salvus allows for flexible model construction, including 3D topography and heterogenous velocity distribution. Model parameters include density (ρ), wave velocities (Vp and Vs), and quality factors (Qp and Qs) to account for wave damping. The model domain is meshed within the software package, using hexahedral elements for 3D simulations and rectangular elements for 2D simulations. In order to facilitate wider accessibility to similar calculations, alternative software options such as OpenSWPC^54^, Seissol^55^, or SpecFEM^56^ offer equivalent capabilities and are open source.

### 3D models

We employ 3D models to investigate spatial effects on wave amplification, while 2D models (see next section) are used to study sensitivities, such as permafrost effects, or azimuthal dependencies on dynamic stress distributions during an earthquake.

**Fig. 1:**
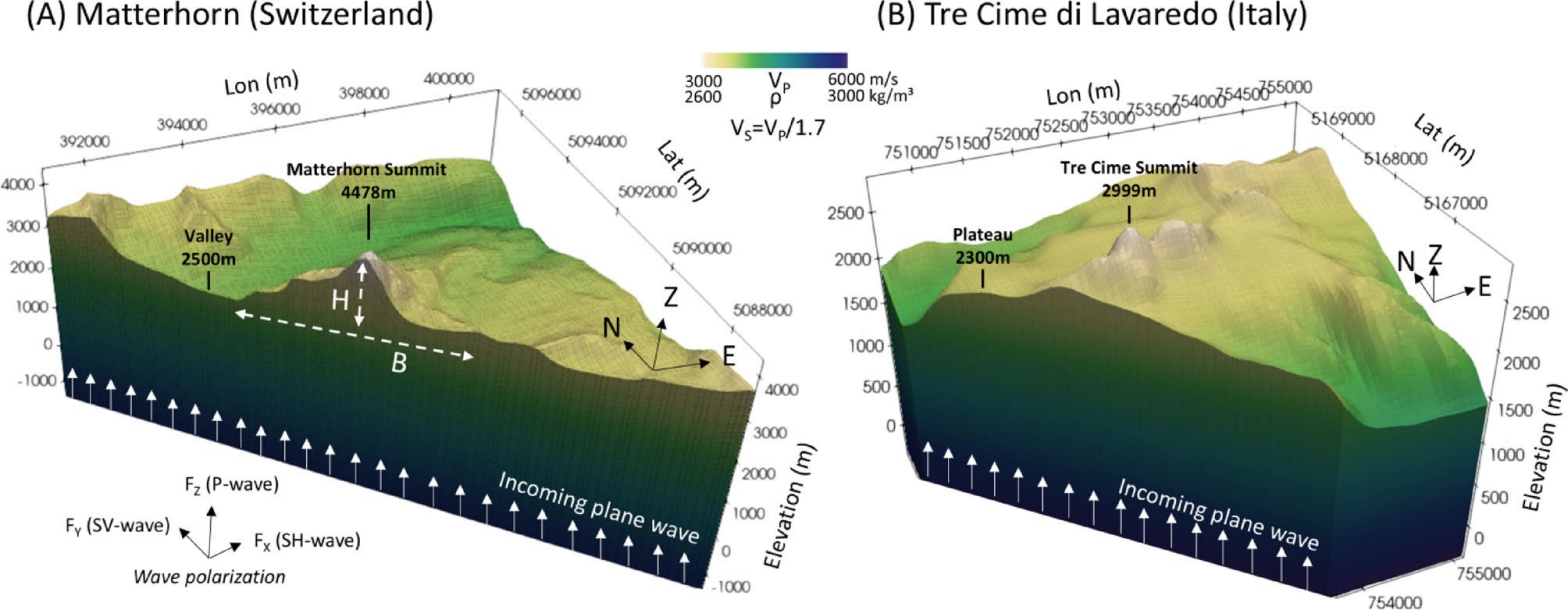
(**A**) Model setup of the Matterhorn and (**B**) Tre Cime die Lavaredo including a high-resolution DEM and velocity gradient. The initial velocity is Vp = 3000 m/s at the summit and increases to Vp = 6000 m/s at a depth of -2000 m. The Q-values (Qp = 200, Qs = 100) describe the attenuation of the seismic waves due to dissipation of energy while the waves travel through the rock. The Q-values are assumed to be constant throughout the model. To simulate the seismic response, a plane wave with a broad frequency spectrum ranging from 0.3 Hz to 3.5 Hz is used. The source wavelet is an Ormsby-function (Fig. S1), and the source mechanism consists of three force vectors in the X, Y, and Z directions, generating S- and P-waves. The dimension of the mountain is described using *H* (valley-summit elevation difference) and *B* (extension of the mountain’s base). Both figures are generated using open-source software package Paraview (version 5.10.1, www.paraview.org).

We utilize a high-resolution DEM obtained from the Federal Office of Topography of Switzerland (Dataset: swissALTIRegio, www.swisstopo.admin.ch) to accurately represent the topographic shape of the Matterhorn in three dimensions. The DEM has an original resolution of 10 m and covers an area of approximately 10 km (x) × 10 km (y) × 6.5 km (z) (Fig. 1A) around the Matterhorn, however, we applied a slight Gaussian smoothing to the DEM to prevent numerical artifacts caused by sharp artificial element intersections. While this may slightly reduce the resolution of small-scale features (as indicated in Fig. 1), their impact on the relatively large wavelengths we are simulating remains negligible. The modeled elevation ranges from -2000 m (below sea level) to the peak elevation of 4478 m. The wavefield and geometrical model are resolved using a minimum of two elements per wavelength and a polynomial order of 4 (~ order of nodes per element). This ensures accurate representation of the wavefield and the geometry of the models with an element size of less than 200 m, discretized by further points, due to high nodal resolution based on the chosen polynomial order. Absorbent layers (not depicted in Fig. 1) are attached to the actual model domain to ensure sufficient wave damping at the model boundaries. In order to analyze a wide range of frequencies, we use an Ormsby wavelet as the source, which has a trapezoidal frequency spectrum that is flat between 0.3 and 3.5 Hz (Fig. S1), to account for the typical frequency range observed in terms of mountain resonances^22,23^. However, to produce the results in Fig. 5 specifically, we used a Ricker-wavelet with the centre frequency fitting the crucial resonance frequencies found for the specific mountain. To simulate the incoming seismic wave from a local or regional earthquake, we model a vertical plane wave excited by three force vectors (in the X, Y, and Z directions) at each cell of the mesh at elevation z = 0. This represents a plane wave excitation with both P- and S-waves. Synthetic receivers across the summit are located along the NS-axis and EW-axis across a length of 5 km at intervals of 100 m.

The subsurface properties of our reference model include a linear velocity gradient, with a velocity of VP = 3000 m/s at the summit and VP = 6000 m/s at z = − 2000 m (Fig. 1). This represents lower velocities near the surface and higher velocities in the crust. The velocity values at the surface are based on the geological composition of the highly weathered and fractured Gneiss in the mountain top region^52,68^, while higher velocities are typically expected in deeper regions with more compressed bedrock. The shear wave velocity VS is always equal to VP/1.7. We assume lateral homogeneity in the velocity distribution. Additionally, the Q-values (QP = 200, QS = 100), which represent the energy dissipation associated with seismic waves, are assumed to be constant throughout the model. Although the used set up underlies simplifications, such a velocity gradient model is suitable to approach the modeling which focuses on topographic effects.

For the Tre Cime di Lavaredo (peak elevation of 2999 m) in Italy (Fig. 1B), we utilize a DEM derived from INGV (Italian National Institute of Geophysics and Volcanology, https://tinitaly.pi.ingv.it/) with a 10-m resolution. Although, a limitation of the topographic model concerns the structural resolution of the three finger-like peaks of the Tre Cime massif. The available DEM does not fully capture the separation of the famous three individual towers (see Supplementary Fig. S5), resulting in a partial merging of these features within the meshing of the numerical model. While this represents a constraint on the fidelity of the topographic representation, we consider the available DEM sufficient for the purposes of this study. Because of a minimum seismic wavelength of approximately 485 m (based on a maximum frequency of 3.5 Hz and a minimum S-wave velocity of 1700 m/s), the model resolution is appropriate for capturing the general wavefield dynamics, amplification behavior and potential resonances of the massif.

In total, the model covers an area of approximately 5 km (x) × 4 km (y) × 3 km (z). The subsurface properties of the Tre Cime di Lavaredo model are based on the same velocity model as the Matterhorn. The mountains in the Dolomites typically show relatively thin stratigraphic layers, which are neglected in our idealized models. Hence, the main distinction between the modeled mountains is their topography, which sets the focus on comparing topographic effects and wave dynamics in our study. Here, the synthetic receivers across the rock formation are located along the NS-axis and EW-axis across a length of 4 km at intervals of 100 m.

### 2D models

While our primary focus is on spatial topographic effects influencing amplification distribution and wave dynamics in 3D models, we utilize 2D models for additional sensitivity studies. These include permafrost effects or stress distribution under non-vertical incoming waves, allowing us to reduce computational costs. The 2D models presented in later sections represent a north–south cross-section of the Matterhorn, spanning approximately 8000 m horizontally and 6500 m vertically. The distributions of velocity, density, and Q-values, along with the plane wave source approximation, are designed to be analogous to the 3D case for direct comparison. The reference 2D model is depicted in the supplements (Fig. S2). Frozen rock, such as permafrost, is typically characterized by higher seismic velocities and denser material due to the presence of ice in fractures and voids^36,37^. To model scenarios with permafrost inside the mountain (used later in Fig. 6), we systematically decrease the amount of permafrost and increase the seismic velocities by up to 50% compared to the reference model depicted in Fig. 1. To account for effects of non-vertical incoming waves, the source excitation vectors (Fig. 1) are rotated 45° around the EW-axis, and the source plane is tilted to approximate P- and S-plane waves incoming from the north or south.

## Results

### Seismic responses of Matterhorn (Switzerland) and Tre Cime di Lavaredo (Italy)

**Fig. 2:**
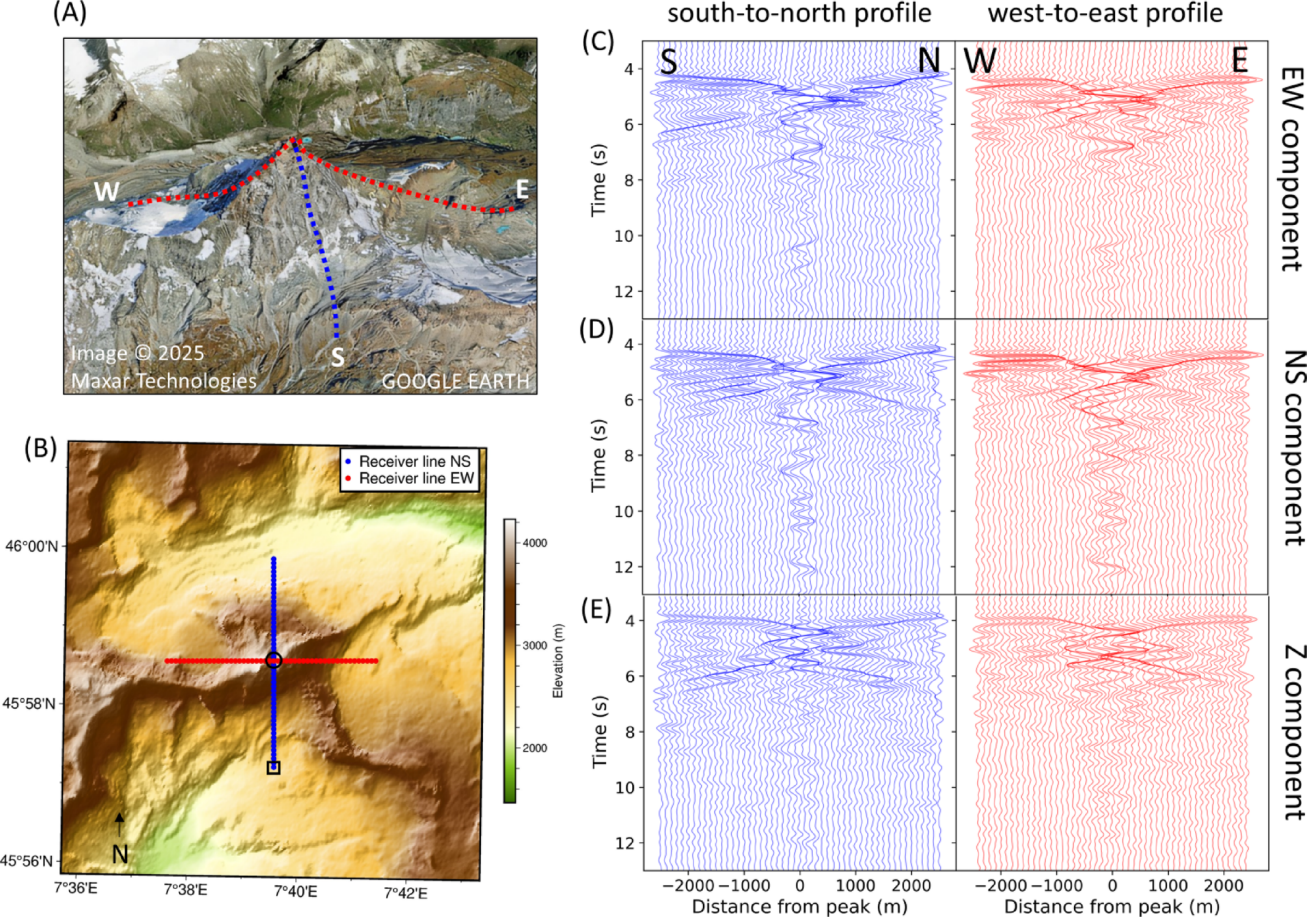
(**A**) and (**B**) Location of synthetic receivers positioned along the east–west (EW, red) and north–south (NS, blue) axes, spanning across the summit for Matterhorn. C-E: Synthetic seismograms extracted along the NS- and EW-axis (left and right), for all three components (from top to bottom). The amplitudes of the seismic traces in each subfigure are normalized to the maximum amplitude of their respective figure. The black circle and rectangle in B correspond to the summit and valley data presented in Fig. 4. Image data in (**A**): Google Earth, accessed March 2025.

Synthetic seismograms are extracted from receivers positioned along the summit of the mountain (Fig. 2A,B). The wavefield (Fig. 2C–E) exhibit higher signal amplitudes at the central locations of the profiles compared to stations situated in adjacent valleys. The central receivers are located at the summit. These prolonged and amplified signals are predominantly observed on the horizontal components of ground motion, particularly in the north–south (NS) direction (Fig. 2D).

The variation between the receivers along the north–south axis and the east–west axis is negligible, suggesting it may be attributed to the azimuthal symmetry of Matterhorn. Resonances with prolonged durations of 10 s are primarily observed on the NS component at the summit. Such resonances and signal amplification can be explained by reflections off the mountain flanks resulting in constructive interferences and reverberations of the waves trapped in the summit of the mountain. Aside the resonances at the central stations, a scattered wavefield can be observed. This wavefield is likely arising from multiple reflections off the sides of the mountain, as well. However, compared to the prolonged resonances, such reflections are not trapped inside the upper part of the mountain.

**Fig. 3:**
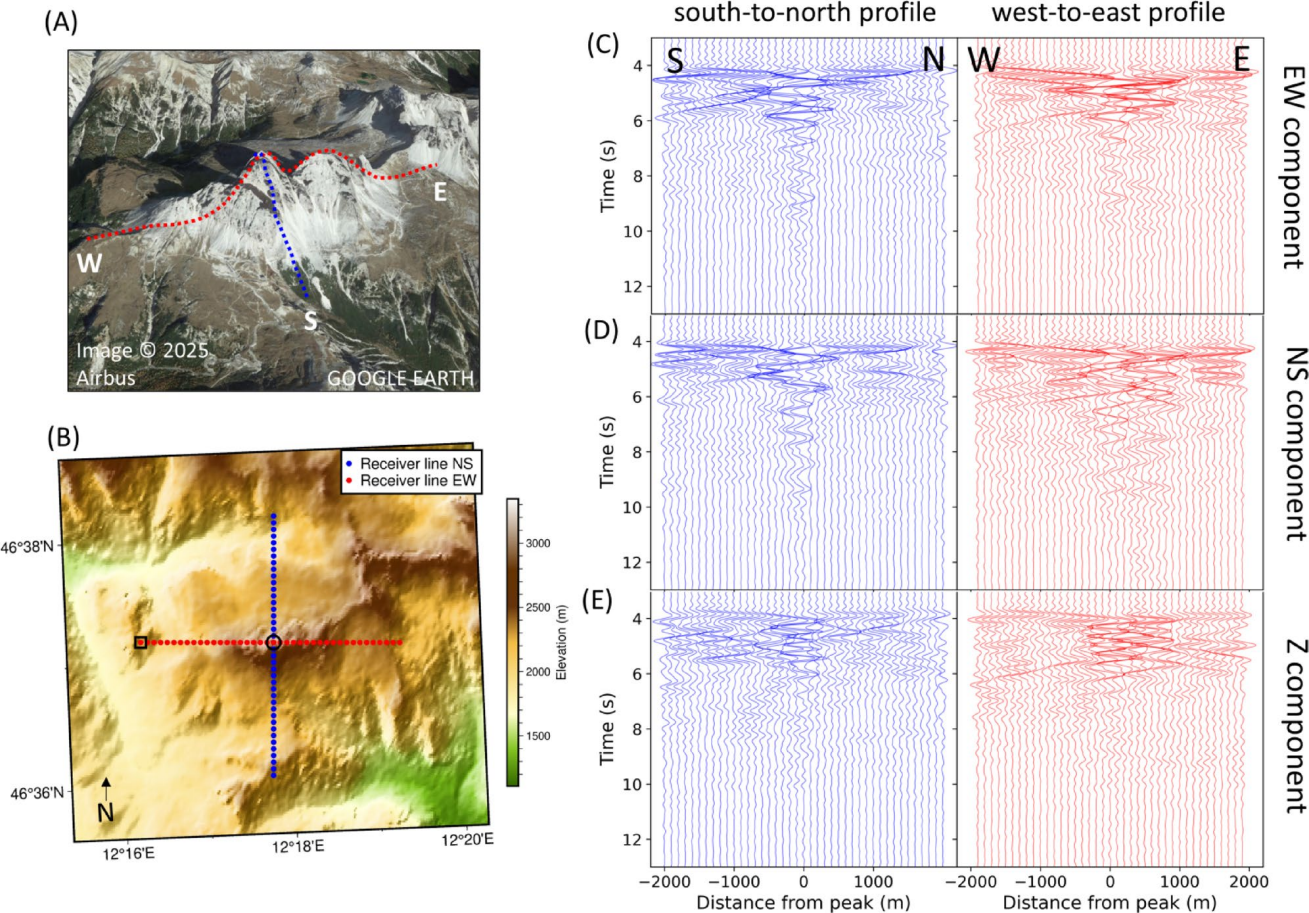
(**A**) and (**B**) Location of synthetic receivers positioned along the east–west (EW, red) and north–south (NS, blue) axes, spanning across the summits of the Tre Cime di Lavaredo. (**C**–**E**) Synthetic seismograms extracted along the NS- and EW-axis (left and right), for all three components (from top to bottom). The amplitudes of the seismic traces in each subfigure are normalized to the maximum amplitude of their respective figure. The black circle and rectangle in B correspond to the summit and valley data presented in Fig. 4. Image data in (**A**): Google Earth, accessed March 2025.

An analogue simulation is performed for the Tre Cime di Lavaredo. Receivers located on the summits exhibit amplified signal amplitudes compared to stations in nearby valleys, both in the north–south (NS) and east–west (EW) directions (Fig. 3C,D). Along the EW-axis, the resonances extend eastwards from the center station due to the extended rock formation of the Tre Cime di Lavaredo (Fig. 3D) in east direction. However, this extension is absent along the NS-profile, indicating significant dependency on the ridge-like geometry of the Tre Cime di Lavaredo. In general, the resonances have a duration of approximately 6 s. Similar to the results for Matterhorn, a weaker scattered wavefield is observed apart from the center stations.

**Fig. 4:**
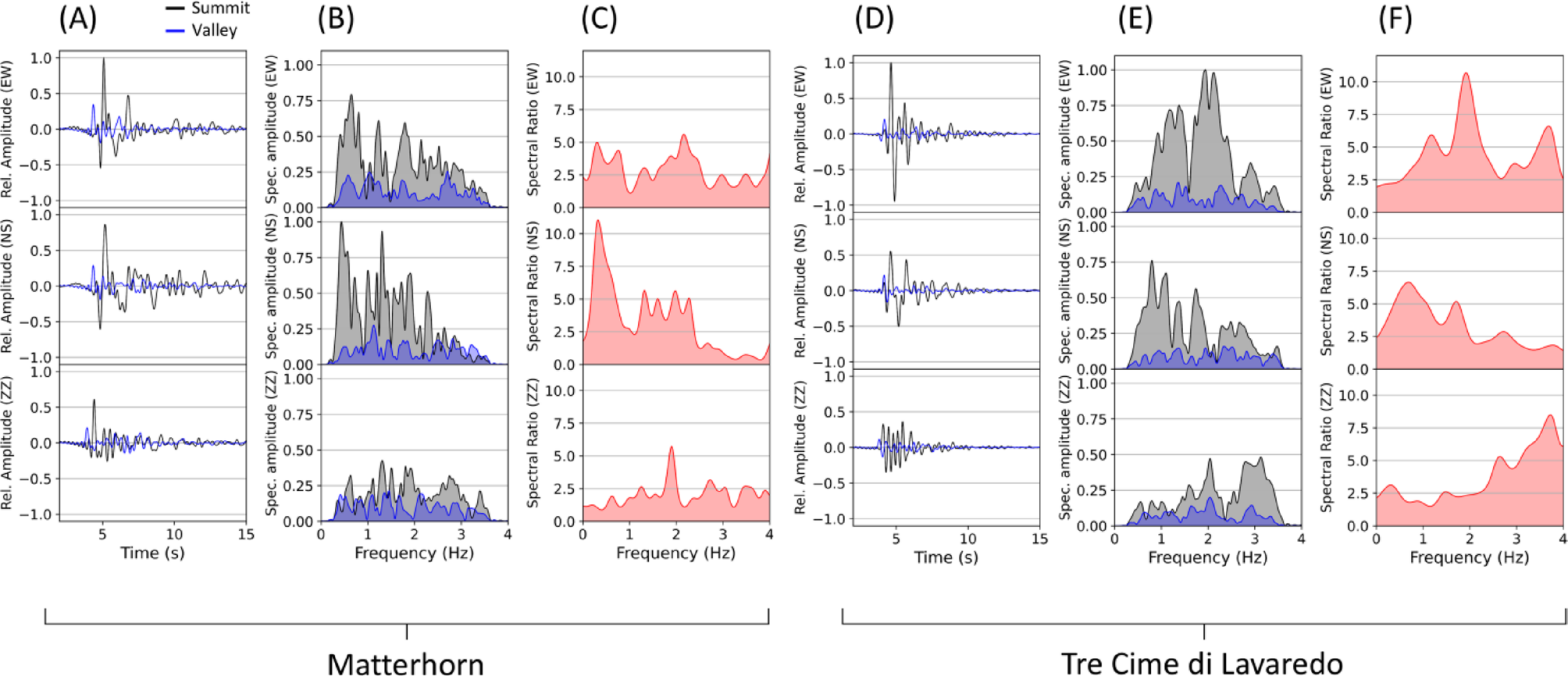
Time series, spectral response and spectral ratio (summit-to-valley) of Matterhorn (**A**, **B** and **C**) and Tre Cime di Lavaredo (**D**, **E** and **F**) to a source wave with frequencies ranging from 0.3 Hz to 3.5 Hz. The black line is the synthetic seismogram extracted at the summit location marked by the circle in Fig. 2 (Matterhorn) and Fig. 3 (Tre Cime di Lavaredo), respectively. The rectangle in Fig. 2 and Fig. 3 marks the location of the extracted (blue colored) data. Dominant amplification and maximum signal amplitudes at the summit occurs primarily on the horizontal components along with distinct frequency peaks, indicating resonance modes.

For comparing frequency dependent amplification between the valley and summit, we compare waveforms, spectra and spectral ratios (Fig. 4). The spectral ratios are derived from dividing the summit spectra by the valley spectra, while the spectra were slightly smoothed to compare major frequency ranges and avoiding bias by strong contrasts of the spectral curves. Regarding the Matterhorn, notable spectral amplification is observed on the horizontal components (Fig. 4A–C), peaking at magnitudes up to eleven times larger than the signal amplitude at a valley station. Distinct resonant frequencies, notably between 0.4 and 2.5 Hz, are discerned, with the most prominent peak occurring at 0.4 Hz on the NS-component (Fig. 4B), along with the highest amplification. A minor peak is at 1.8 Hz on the EW-component. Conversely, vertical amplitudes are comparatively subdued, underscoring a horizontal seismic energy amplification.

Concerning Tre Cime di Lavaredo, horizontal components similarly display substantial amplification, with signal magnitudes reaching approximately six to ten times larger amplitudes compared to valley station signals of the same frequency (Fig. 4C,D). Again, vertical amplitudes are reduced relative to horizontal motions. Noteworthy resonant frequencies of 0.8 Hz and 1.0 Hz (NS-component and EW-component), 2.0 Hz (EW-component), and 3.0–4.0 Hz (Z-component) are identified (Fig. 4D). However, the most prominent peak is at approx. 2.0 Hz, along with the highest amplification. Distinct frequency peaks persist up to of 3.0 Hz. This excitation of higher frequencies at Tre Cime di Lavaredo can be attributed to the comparatively small size (*H* = 600 m, *B* = 2000 m) in contrast to the Matterhorn’s dimensions (*H* = 2000 m, *B* = 5000 m). Interestingly, the lower frequency peak of 0.8 Hz is dominant on the NS-component (perpendicular to the EW oriented rock formation), while the higher frequency peak of 2.0 Hz is dominant on the EW-component. This indicates combined dependency of the resonance modes on motion direction, wavelength as well as summit geometry and extension.

**Fig. 5:**
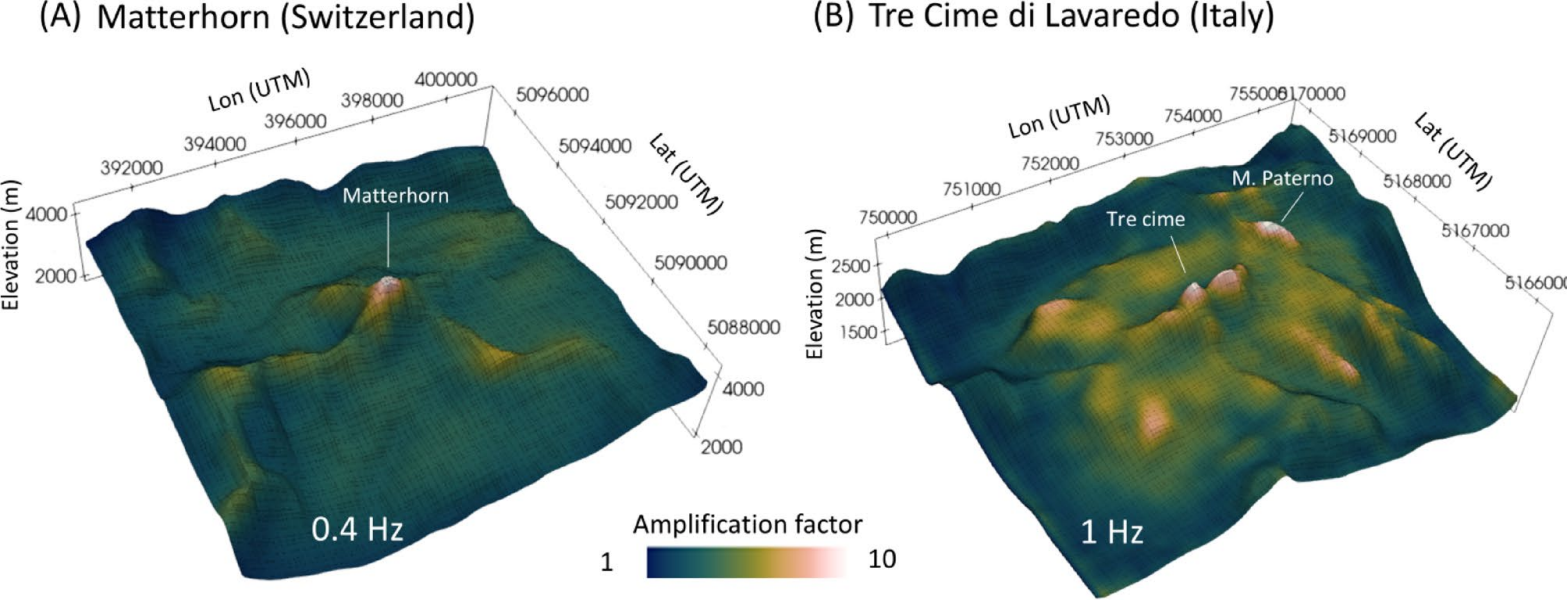
Distribution of amplification factors for the region at Matterhorn (**A**) and Tre Cime di Lavaredo (**B**). The dominant frequency of the source Ricker-wavelet is 0.4 Hz for Matterhorn and 1.0 Hz for Tre Cime di Lavaredo, based on findings from the spectral analysis. The thin black lines represent the mesh. Here, the amplification is calculated from the ground motion’s (EW, NS, ZZ) magnitude. The values (color scale) are normalized to the respective minimum. Both figures are generated using open-source software package Paraview (version 5.10.1, www.paraview.org).

Through computed maps showing the spatial distribution of amplifications factors (Fig. 5), we elucidate the maximal ground motion amplification at Matterhorn, as well as Tre Cime di Lavaredo. The analysis of the response to a 0.4 Hz wave frequency reveals widespread peak values across the Matterhorn summit (Fig. 5A), attributable to the elongated wavelength of the vertically incoming P- and S-wave. Adjacent summits and ridges experience significantly lower excitation from the seismic event. However, the amplitude of higher-frequency waves (e.g. 1.0 Hz) may be amplified by smaller mountains, as shown for the Tre Cime di Lavaredo (Figs. 4C,D, 5B). At the Tre Cime di Lavaredo, notably elevated amplification factors manifest at the three summits of Tre Cime di Lavaredo and Monte Paterno, situated eastward. The plateau of the mountains exhibits a modest amplitude increase as well, indicating larger scale amplification. In addition to the three-dimensional maps, we study further details using highly resolved 2D models in the next sections.

### The effect of permafrost on signal amplification

**Fig. 6:**
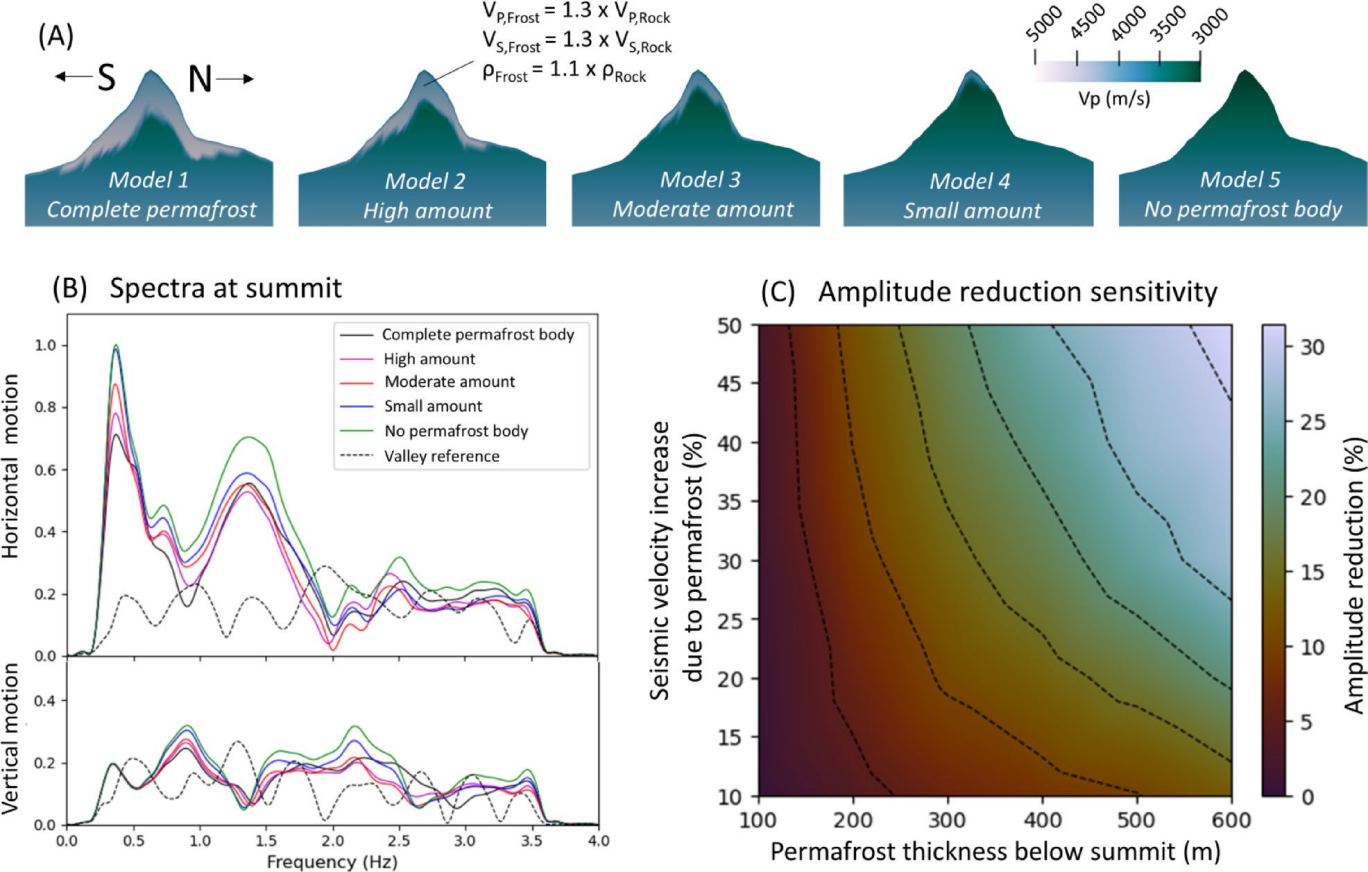
(**A**) Two-dimensional models of Matterhorn including different amount of permafrost inside the mountain. (**B**) Spectra of the vertical and horizontal components, extracted from the synthetic receiver atop the summit. The dashed black line represents the spectrum observed at a receiver located in the valley. C: Sensitivity of the amplitude reduction in terms of permafrost thickness and seismic velocity increase compared to a model without a frozen layer in the rock. The dashed contour lines are isolines (5% steps).

We assess the potential impact of permafrost on seismic amplifications by postulating heightened density and wave velocity within a certain subsurface layer inside the mountain, modeled by an ice shield below the surface (Fig. 6A). This model serves as an idealized approximation based on the thermal model conducted by Noetzli and Gruber^57^ and permafrost distribution inside mountains described in Arenson et al.^58^. In our model, the permafrost body’s dimensions systematically decrease in thickness and extension towards the valley, assuming that the melt starts at lower altitudes. The bedrock below the ice shield is assumed to be ice-free. Five models are utilized for our study. Model 1 presupposes an idealized large permafrost body inside the mountain (thickness below the summit is 600 m). Models 2–4 represent diminishing ice bodies, while model 5 represents the mountain without any frozen material in the rock, resulting in reduced density and velocity due to air or water occupying fractures, voids, and cracks.

Spectral analyses are performed for the synthetic receiver situated atop the summit, revealing two prominent peaks at approx. 0.4 Hz and 1.4 Hz (Fig. 6B), consistent with simulated peaks derived from a three-dimensional model (Fig. 4B). As permafrost diminishes, spectral amplitudes at 0.4 Hz and 1.4 Hz decrease. This decrease is significantly more pronounced at 0.4 Hz than at 1.4 Hz. For frequencies 2–3.5 Hz, the effect on the amplification is negligible. However, depending on the permafrost distribution and mountain geometry, e.g. in case of smaller-scale sizes such as Tre Cime di Lavaredo, higher-frequencies could be affected as well in other scenarios, as shown in Fig. 4. Similar to previous results, the effect is notably enhanced for horizontal motions compared to vertical motions (Fig. 6B). We performed a sensitivity study, which involves 30 models with different combinations of permafrost thickness (varied between 100 and 600 m) and seismic velocity increase, caused by frozen water in the permeated rock (varied between 10 and 50% increase). It reveals that the maximum reduction (~ 30%) of amplitude amplification occurs in case of a thick and dense permafrost layer (see Fig. 6C). This suggests that both a decrease in the thickness of the ice shield and a decrease in the amount of ice within the shield itself lead to higher PGV values, while the sensitivity is slightly higher for the ice thickness than the velocity increase. However, it is unlikely that very small ice bodies have the potential to significantly impact the wavefield with frequencies less than 3 Hz.

One explanation for this reduction of amplification is the contrast in material properties between frozen layers and unfrozen layers within the mountain. When seismic waves encounter the rock-ice discontinuity, they are partly reflected. In addition, the transmitted waves are less likely to be trapped in the summit due to their elongation of the wavelength when entering a material with higher seismic velocities. This results in a more efficient transportation out of the permafrost region after reflections at the free surface, thereby preventing the generation of resonances. Another potential reason is the increase of mass by filled voids of frozen rock compared to thawed rock, which likely lowers the amplitude of the vibrations.

### Dependencies on earthquake azimuth and incidence angle

**Fig. 7:**
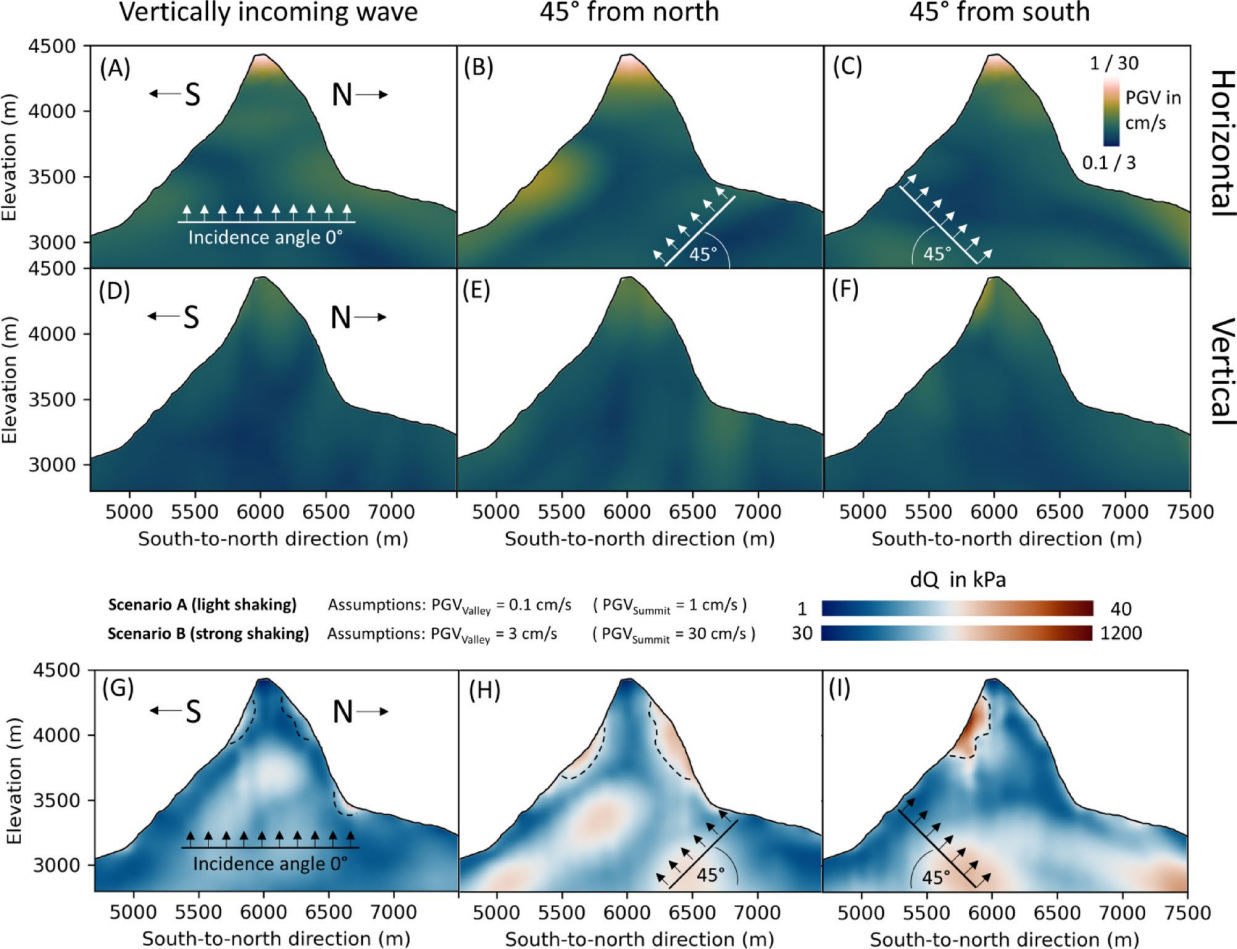
Maximum horizontal (**A**–**C**) and vertical (**D**–**F**) ground motion amplitude and maximum deviatoric stress dQ (G-I) across all time steps during the simulated earthquake. An earthquake in the north and south of Matterhorn is modeled using an incident plane wave with an angle of 45°. The black arrows denote the P-wave propagation direction. The polarization of the S-wave is perpendicular to the arrows. The values are commonly scaled to show the difference between the amplitudes of the vertical and horizontal motion. Scenario A and B (in the title of G-I) provide estimates of absolute PGV and stress values, based on PGV observations during an earthquake provided by Cauzzi et al.^59^.

In this section, we analyze the seismic waves approaching the Matterhorn from the north and south directions using a two-dimensional cross section. We consider non-vertical incidence angles and use an angle of 45° in both cases (north and south). These models serve as the foundation for the simulation results presented in Fig. 7, which is why we focus on two dimensions in this section. We find that, consistent with the three-dimensional model (Fig. 5), the maximum amplitude occurs at the summit in the horizontal component (NS-direction) in all three cases (0° vertical incidence, 45° south, and 45° north). In the case of a vertical incoming plane wave, the amplitudes at the northern and southern mountain flanks are similar due to the symmetry of the mountain and the incident wave. However, when waves approach from the north and impact the southern parts of the formation, the horizontal ground motions in the valley to the south of the Matterhorn slightly increase (Fig. 7B). As a result, the contrast between maximum amplitudes at the summit and amplitudes in the southern valley slightly decreases. Conversely, when the wave arrives from the south, the horizontal signal amplification is comparable to the results obtained with a vertical polarization of the incoming wave (Fig. 7C). Interestingly, the vertical ground motion is slightly increased at the very steep part of the southern flank (> 4200 m). Although the directional characteristics of the wave influence the distribution of amplification factors, the mountain experiences the maximum oscillation at the summit in horizontal directions under all circumstances, which supports the findings depicted in Fig. 4.

### Dynamic stress changes during an earthquake

In order to identify locations of potential slope instabilities during an earthquake, we explore the linkage from seismic ground motion (in Fig. 7) to the consequential stress distribution. Thus, the distribution of the dynamic stress field is calculated. The underlying deviatoric stress, denoted as $dQ$, refers to material deformation (e.g., by seismic waves) without a change in volume of the material. Hence, the initial hydrostatic stress is subtracted from the deviatoric stress. Using the second invariant ${J}_{2}$ of the two-dimensional total stress tensor ${\sigma }_{ij}$ derived from the numerical simulation, the scalar of the deviatoric stress $dQ$ can be expressed by

1 $$dQ=\sqrt{{J}_{2}}=\sqrt{\frac{1}{2}\left({\left({\sigma }_{xx}+P\right)}^{2}+{\left({\sigma }_{yy}+P\right)}^{2}{+ \left({\sigma }_{zz}+P\right)}^{2}+2{\sigma }_{xy}^{2}\right)}$$

using the hydrostatic stress (or pressure)

2 $$P=- \frac{1}{3}\left({\sigma }_{xx} +{\sigma }_{yy} +{\sigma }_{zz}\right)$$

Although the stress calculation is performed in two-dimensions, the out-of-plane stress ${\sigma }_{zz}$ must be considered. Note that the z-axis is directing out of the x–y-plane and not in vertical direction. The detailed derivation of the formulation is provided in the supplementary material. By examining the peak dynamic stress per mesh element across all simulation time steps, we can ascertain the stress distribution throughout the two-dimensional model of Matterhorn.

While the summit of the Matterhorn experiences the highest magnitude of motion (Fig. 7), the peak stress at the surface manifests on the mountain’s flanks (Fig. 7). For vertically incident waves, a slight elevation in stress is observed on the southern flank, particularly within elevations spanning approximately 3800 m to 4300 m, while the northern flank exhibits comparatively weaker stress variations (Fig. 7G). Moreover, an escalation in stress is simulated at the topographic salient point situated on the northern side of the mountain, at an altitude of approximately 3400 m.

In case the wave arrives from north, the stress is significantly increased compared to a vertical oncoming wave. The location of increased stress on the southern flank is shifting to lower altitudes and covers a larger area. Conversely, the stress on the northern flanks intensifies strongly in magnitude and encompasses a significant broader area, if the waves are approaching from the north, (Fig. 7H). An explanation could be surface waves from north, that are generated by the incident incoming wave. A wave coming from the south leads to a strong increase in stress on the southern flank at an elevation higher than 3700 m (Fig. 7I). Interestingly, there is no increase in stress observed on the northern flank in this scenario. Overall, a non-vertical incoming wave generally leads to broadened areas of increased stress on mountain flanks, although minor effects of the valley-to-summit amplification. The variation in results for waves approaching from the south and north suggests that the distribution of stress on the mountain flanks depends on the azimuth of the earthquake source and on the specific topographic effects of the mountain. Our findings suggest a combined effect of the wave front’s incoming direction and the mountain’s geometry. When the wave enters at a non-vertical angle, it becomes significantly trapped within the mountain, leading to strong wavefield interferences and, consequently, higher deviatoric stresses. In contrast, a vertically arriving wave exhibits weaker trapping effects, resulting in less pronounced deviatoric stress intensities. Nevertheless, an increase of deviatoric stress at the southern flank of Matterhorn is derived in every scenario.

To obtain absolute stress estimates, we consider two potential scenarios involving PGV values at the Summit. According to the research conducted by Cauzzi et al. in 2017^59^, during a magnitude 4.4 earthquake near Matterhorn in Vallorcine in 2005, the PGV values at the surface ranged from approximately 0.1 cm/s for light shaking to a maximum of 3 cm/s for strong shaking, close to the epicenter. Considering these findings, we assume Scenario A with a peak amplitude of 1 cm/s at the summit (ten times higher than 0.1 cm/s in the valley), and Scenario B with values of approximately 30 cm/s at the summit (and 3 cm/s in the valley). Incorporating these assumptions, we can conclude that the maximum deviatoric stress levels at the mountain’s flank can reach approximately 40 kPa during light shaking and can exceed 1 MPa during strong shaking (Fig. 7).

## Discussion

We simulated the resonant seismic response of Matterhorn and Tre Cime di Lavaredo using full waveform modeling and demonstrated seismic amplifications at the summits of the mountains. These amplifications depend on the frequency of the incoming wave, the potential presence of permafrost, as well as the geometrical characteristics of the rock formation. Although the reasons for rockfalls might be due to a combination of multiple factors (e.g., heavy rainfall, melting permafrost, preexisting fractures), short-term dynamic stress changes could also play a significant role in triggering slope instabilities^60,61^. Mainly earthquakes with a large magnitude trigger landslides immediately; however, frequent seismicity with small-magnitude earthquakes can destabilize the rock progressively^61,66^, resulting in delayed slope failures. Hence, we furthermore analyzed the dynamic stress variations during an earthquake to identify locations of elevated hazard in terms of slope instabilities (Fig. 7). Our results underscore the significance of incorporating realistic topographic models in conjunction with wave characteristics and stress computations.

Although the general database of seismological recordings at mountains is limited, the findings of our study mostly confirm the observations from existing previous studies that have investigated the effects of steep topography on seismic amplification. In the following, we point out the most important comparisons.

**Fig. 8:**
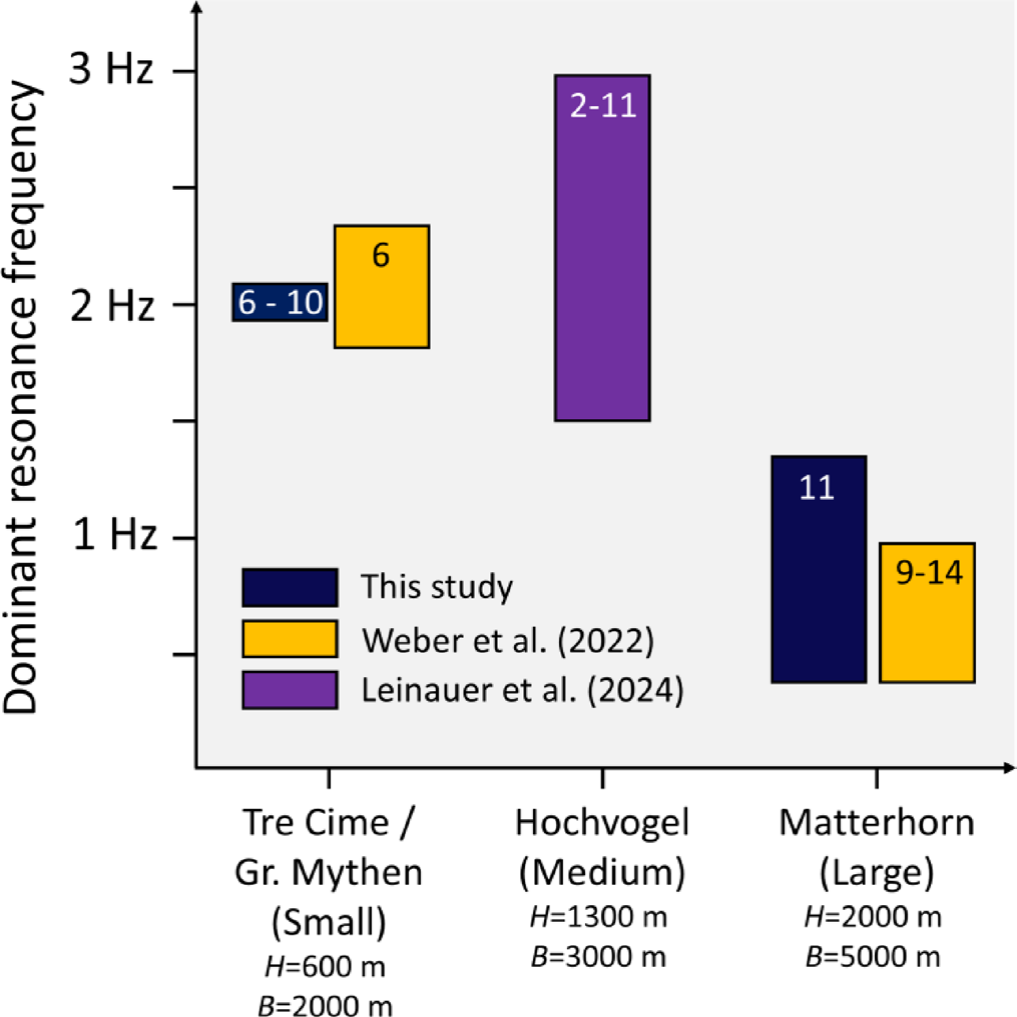
Comparison of dominant resonance frequencies (span of the bar) and average amplification factors (numbers in bars) from existing studies and our findings. The four studied mountains are sorted according their size, from small to large (approximated height H and extension B, see Fig. 1). Note that the results from Weber et al.^23^ and Leinauer et al.^22^ are based on continuous ambient noise measurements and not specifically on earthquake signals.

Weber et al.^23^ conducted an experimental study at the Matterhorn and performed numerical modal analysis to identify distinct resonance. Their observations highly support our model results of mountain-specific resonances at the Matterhorn at frequencies around 0.4 Hz (Figs. 4B and 6A), with dominant motion in horizontal directions, especially in the NS-direction. However, we additionally can identify resonances at 1.4 Hz and a minor peak at 1.8 Hz, which can be explained by signal modulations due to reflections inside the mountain summit. Weber et al.^23^ analyzed ambient noise, which might not reflect the full spectrum of potential resonance modes and frequency-dependent amplifications that could be additionally excited by earthquakes. Nevertheless, they detected noise amplifications of 9 times on average and 14 times as a maximum at the summit of Matterhorn, comparable to simulated amplifications (~ 10 times) in our study. In addition to Matterhorn, they measured resonances of 1.8 Hz and 2.3 Hz at the mountain Großer Mythen, which is considerably smaller in size. These findings validate the presence of higher frequency resonances that depend on the mountain’s volume and dimensions, as we demonstrated through the comparison of synthetics derived from models of Matterhorn and the smaller Tre Cime di Lavaredo. Moreover, Leinauer et al.^22^ performed seismic measurements at a mountain flank of the Hochvogel (Austria) and suggest an amplification of ground motion due to topographic effects of a factor of 2–11, which further supports the estimations based on our models. A comparison of resonance frequency and amplification factors found in existing studies and our study are depicted in Fig. 8, indicating the tendency of higher resonance frequencies and lower amplification factors for smaller mountains. This finding coincides with the results derived from measurements presented by Kleinbrod et. al.^64^. The relatively broad frequency range of resonances between 1.5 and 3.0 Hz observed at Hochvogel in Austria^22^ could be explained by the less distinctive mountain shape compared to Matterhorn, Tre Cime di Lavaredo, or Großer Mythen. This likely extenuates the generation of distinct resonances due to less freedom of oscillation.

The findings of Massa et al.^24^, who conducted a comprehensive review of topographic effects based on experimental data collected over four decades, confirm predominant horizontal motions and found amplifications factors of 2–4 for common mountain geometries. The amplification factors derived by our numerical simulations are significantly higher (up to 10), which underscores the significance of highly elevated amplification in case of extreme mountain shapes. Furthermore, they observed that wavefields tend to be polarized perpendicular to the main orientation of the topographic formation. This supports our observation of major lower-frequency motion in the NS-direction at the Tre Cime di Lavaredo, which has a main rock formation orientation in the EW direction (Fig. 3). In addition to the NS- and EW-oriented profiles across the Matterhorn, we conducted a further simulation placing receivers along the ridge-like formation surrounding the Matterhorn massif (see Supplementary Fig. S4). As indicated by our findings in Fig. 5A, signals are amplified along the ridges, though the highest amplification occurs at the summit. These synthetic wavefields align with the structural effects simulated at Tre Cime di Lavaredo. Hence, not only high and steep mountain summits pose hazards of wave amplification, also ridge-like formation lead to moderate amplitude enhancement, as shown by the modelling.

Regarding the influence of permafrost on seismic site response, our study is consistent with the findings of Yang et al.^32^, who used 2D models to investigate the effect of horizontal permafrost layers. They concluded that permafrost can alter site effects, specifically attenuating wave fields. We studied permafrost effects in a steep slope mountain region and demonstrate the importance of considering secondary effects of degrading ice in the mountain that reduce seismic amplifications (Fig. 6). In summary, we find that the presence of permafrost can decrease signal amplifications by up to 30%, based on the studied scenarios. In addition to the increased likelihood of slope instabilities caused by melting ice in the mountains, there is also a heightened amplification of earthquakes. This means that alpine regions face an even greater risk if permafrost degradation continues due to ongoing climate warming, posing a significant hazard in the coming decades^62,63^.

Poursartip et al.^30^ and Shen et al.^31^ used generic 2D models of valleys and hills to examine the effects of topographic irregularities and wave incidence angles on ground motions. They found that the incidence angle influences the amplification; however, the magnitude of amplification depends on the topographic feature dimensions and wave frequency. If the wave is efficiently trapped within the feature, amplifications tend to be significantly heightened^30^, which is shown by our models as well. Furthermore, our findings show that the incident angle does not necessarily lead to significantly increased amplification of signals with relatively low frequencies (< 2 Hz). However, we provide evidence that the locations characterized by strong stress changes during an earthquake are influenced by the azimuth and incidence angle (Fig. 7), a factor that was not addressed in the aforementioned studies.

As a further possible application, we evaluated a larger three-dimensional model of Matterhorn to incorporate additional stress dynamics at the summit, which may be missing in two dimensions. This model, shown in the supplements in Fig. S3, demonstrates the dynamic stress linked to the previously discussed wave amplitude amplification results in Fig. 5A. We observe heightened stress on both the southern and northern flanks of Matterhorn, consistent with the two-dimensional stress patterns presented in Fig. 7. However, the three-dimensional stress is largely distributed across the flanks, e.g., as seen in the shifted location of increased stress on the southern flank to the lower southeastern ridge of the mountain, which is not captured using a 2D model.

The seismic response and associated dynamic stress changes during an earthquake are influenced by earthquake source characteristics, such as the angle at which the earthquake strikes, the azimuth of the epicenter, the primary frequency of the seismic waves, and the overall magnitude and mechanism of the event. As a result, different mountains may experience different impacts depending on their sensitivity to higher-frequency local earthquakes, or to regional and teleseismic events. Thus, the findings in our study suggest mountain-specific case studies and experiments to obtain reliable estimates.

The simplified lateral homogeneous velocity gradient used in our models serves as a first approximation of a mountain’s velocity model. In addition to topographic effects on the wave dynamics, internal structures, such as stratigraphic layers and faults, can affect the wavefield through scattering and reflections and have further influence on slope instabilities. Kleinbrod et al.^64^ demonstrated that the seismic response of unstable rock slopes is governed not only by external geometry but also by internal fracturing and heterogeneity, identifying distinct behaviors between depth-controlled and volume-controlled instabilities. Their findings indicate that features such as open fractures and anisotropic material properties can affect wave propagation, amplification, and resonance, which cannot be adequately captured by overly simplistic models. Similarly, Gischig et al.^66^ showed through numerical modeling that internal damage, material contrasts, and pre-existing fractures within the slope strongly influence seismic amplification and displacement, having an additional impact to topographic effects^67^. Moreover, the overall dynamic stress distribution at the mountain flanks during earthquakes, associated with internal fractures and heterogeneities, potentially contributes additional dynamic stress on top of the stresses induced by topographic effects only. Considering these factors, distinguishing between topographic amplification and effects caused by internal heterogeneities would be generally meaningful. While the inclusion of internal structures and local surficial fractures into the model is beyond the scope of this study, these factors should be considered in future research by incorporating these effects along with extreme mountain geometry. However, the resonance frequency of a such huge rock formation is rather controlled by its geometry, while the overall amount of amplification and dynamic stress might be additionally influences by rock properties and structural aspects of the interior.

We have employed either an Ormsby or Ricker wavelet to model the earthquake wave and seismic responses of the mountains. While real earthquake waves exhibit longer wave trains and a broader frequency spectrum, the chosen wavelets are often used to approximate P- and S-wave excitation in seismological modeling. Gischig et al.^66,67^ utilized real earthquake waveforms as boundary conditions for numerical modeling of slope instabilities, employing generic and idealized 2D models. In contrast, the full waveforms generated in our simulations could serve as synthetically derived boundary conditions, inherently accounting for topographic effects.

Numerous studies, as provided in the introduction, have developed approaches to estimate seismic amplification factors and slope instabilities for typical topographic geometries. The simulations performed in this study extend these findings by investigating amplification in extreme, highly slender, and isolated landforms. As a result, the applicability of our findings to more typical alpine mountains is limited, since the findings can hardly be generalized. Hence, we recommend site-specific experimental and numerical investigations in regions characterized by exceptional topographic features with upper-limit amplification effects, which in turn are often insufficiently represented in more generalized approaches.

For future applications, more complex velocity structures including stratigraphic layers along with smaller-scale fractures and faults in different geological settings should be studied to account for further geology-dependent effects. Most importantly, simulation results should be verified and calibrated by observational data collected at various mountains^22,23^, which improves the estimation of absolute PGV and stress values. Due to extreme terrain and inaccessibility, seismological data recorded at steep and high mountains is rare. This challenges reliable estimations of the seismic response of a mountain as a function of earthquake characteristics, e.g., magnitude, focal mechanism, and azimuth. Hence, a broader database with mountain-specific seismic characteristics would be valuable for future studies.

## Conclusion

In our study, we used numerical models along with detailed DEM to simulate wavefield propagation and dynamic stress changes in specific mountains induced by seismic waves from earthquakes.

We modeled mountain-specific responses with distinct resonance frequencies, such as the major peaks at 0.4 Hz at the Matterhorn and 2 Hz at Tre Cime di Lavaredo, two landforms with exceptional geometries. The dimensions and extensions of rock formations influence the frequencies and locations of amplifications. This is evident in the case of Tre Cime di Lavaredo, where amplification occurs along the formation extension in the east–west direction, with higher frequencies due to smaller rock formations. We also found that dominant amplification occurs on horizontal components, which verifies the findings of previous studies. For the first time, the effect of permafrost inside the mountain on wavefield propagation is modeled. We found that a permafrost body reduces the amplifications in the summit by up to 30%, indicating a higher hazard in case of permafrost degradation in the coming decades. However, this effect is frequency-dependent and varies with the thickness and amount of frozen material within the mountain. The increased dynamic stress induced by earthquakes at the mountain suggests locations on the mountain flanks that indicate a higher probability of slope instabilities during an earthquake. Furthermore, the changes in stress distribution depends on topography, wave incidence and azimuth, resulting in significantly increased stress at the flank oriented towards the seismic source.

We are hopeful that our study will help to enhance experimental and numerical hazard assessments of earthquake-induced rockfalls and landslides, as the likelihood of these events is expected to increase worldwide in the future.

## Electronic supplementary material

Below is the link to the electronic supplementary material.

Supplementary Meterial 1

Supplementary Material 2

## Data Availability

The simulations scripts and datasets generated and analysed during the current study are available from the corresponding author on reasonable request.
