# Supplementary material for: Modeling earthquake-induced wavefields and stresses in alpine mountains with extreme topography
Source: Sci Rep. 2025 Jul 4;15:23914. doi: 10.1038/s41598-025-08218-5 (PMC12227684; doi:10.1038/s41598-025-08218-5)
Supplement: Supplementary file 1 — Supplementary Meterial 1 [file 41598_2025_8218_MOESM1_ESM.docx]

Supplementary material to

Modeling earthquake-induced wavefields and stresses in alpine mountains with extreme topography

Fabian Limberger^1*^, Georg Rümpker^1,2^, Jan Philipp Kruse^1^ and Thibault Duretz^1^

^1^Institute of Geosciences, Goethe-University Frankfurt, 60438 Frankfurt am Main, Germany
^2^Frankfurt Institute for Advanced Studies, 60438 Frankfurt am Main, Germany

*Correspondence to*: Fabian Limberger ([f.limberger@geophysik.uni-frankfurt.de](mailto:f.limberger@geophysik.uni-frankfurt.de))


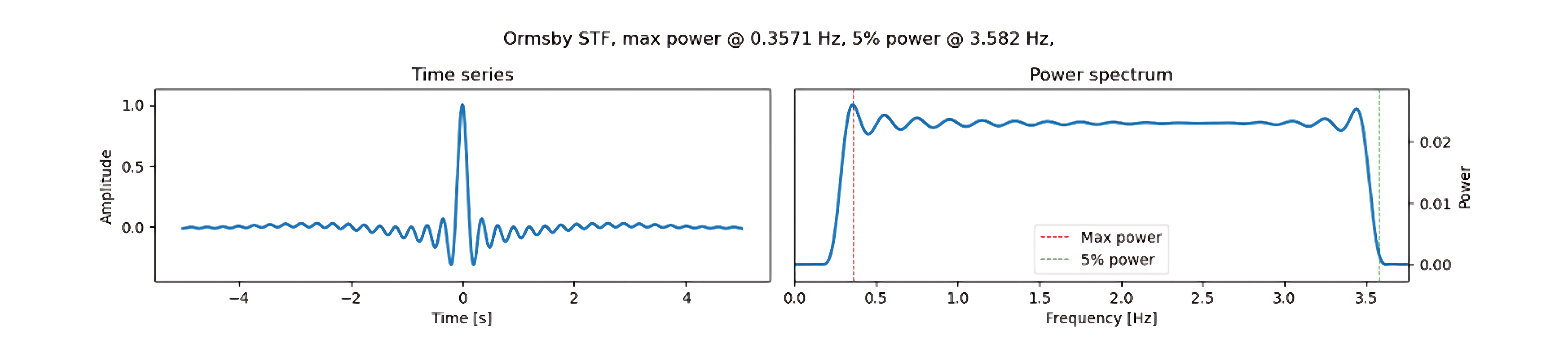


**Figure S1: Ormsby wavelet, which is used as a source time function. The spectra is flat between 0.3 Hz and 3.5 Hz.**

**
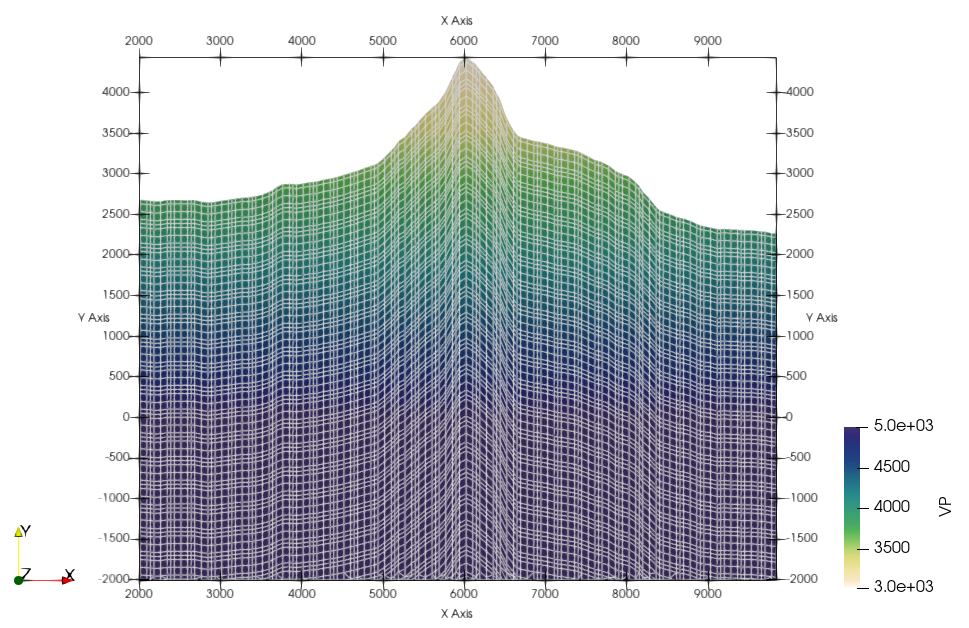
**

**Figure S2: 2D model of the Matterhorn cross section used for permafrost and stress simulations. The thin white lines represent the mesh. This figure is generated using open-source software package Paraview (version 5.10.1, www.paraview.org).**


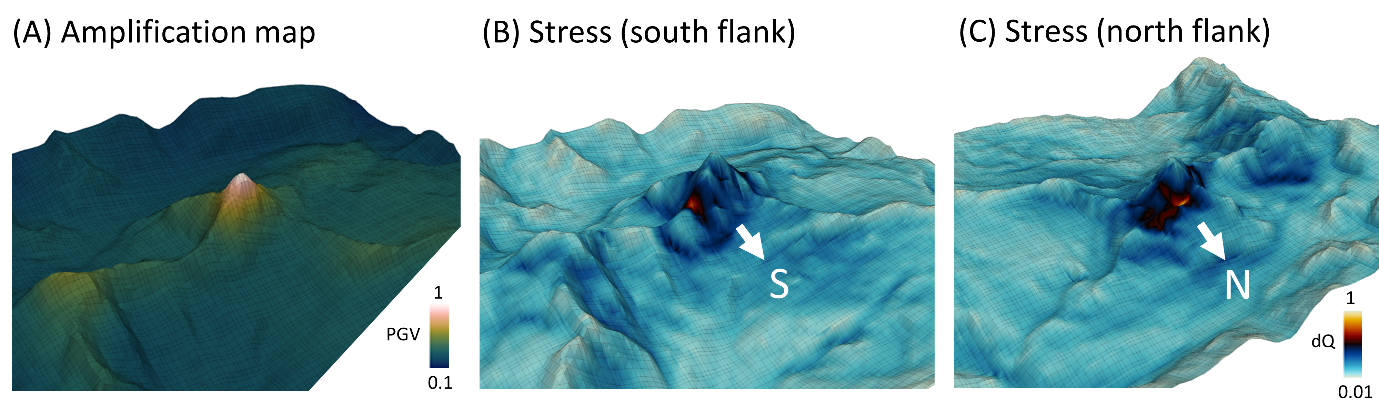


**Figure S3: Maximum PGV (A) and deviatoric stress** $\mathbf{dQ (B and C)}$ **across all time steps during the simulated earthquake in three dimensions. The incoming wave is a vertical plane wave with a dominant frequency of 0.4 Hz (according to Fig. 5A). The values (color scale) is normalized to the respective maximum.** **All figures are generated using open-source software package Paraview (version 5.10.1, www.paraview.org).**


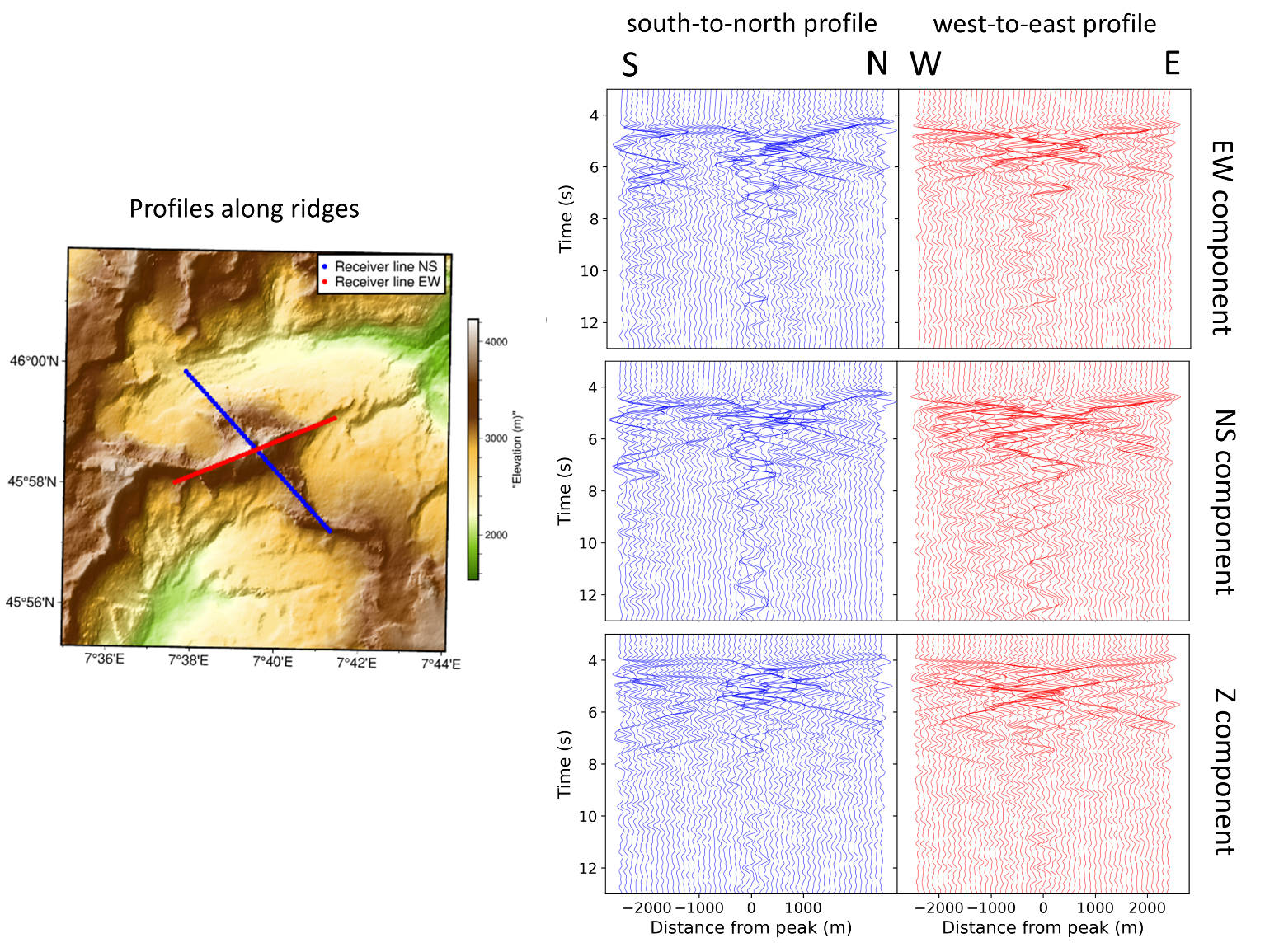


**Figure S4: Left: Location of synthetic receivers positioned along two profiles, spanning across the summits of Matterhorn, along the ridge-like structures. Right: Synthetic seismograms extracted along the NS- and EW-axis (left and right), for all three components (from top to bottom). The amplitudes of the seismic traces in each subfigure are normalized to the maximum amplitude of their respective figure.**


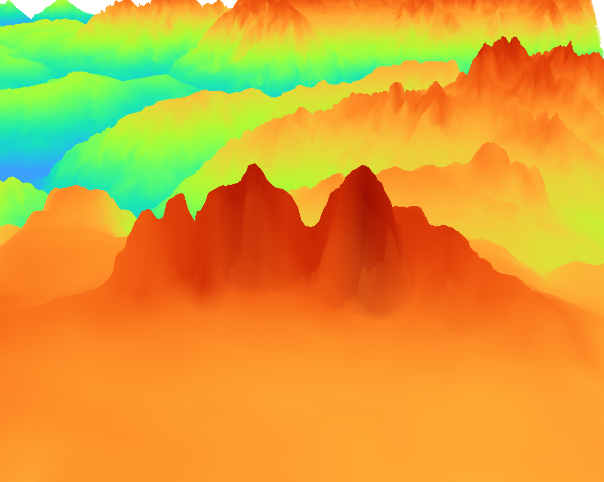


**Figure S5: Raw DEM shows limited resolution of the topographic structures of Tre Cime die Lavaredo. This leads to a merging of the bottom of the three fingers while meshing, as described in the manuscript.**

**In the following, the governing formulations to calculated the deviatoric stress are listed:**

The isotropic stress-strain relation (with plain strain $\epsilon_{xx}=0$) is

$$\left( \begin{matrix} \sigma_{xx} \\ \sigma_{yy} \\ \sigma_{zz} \end{matrix} \right) = \left( \begin{matrix} K + \frac{4G}{3} & K - \frac{2G}{3} & K - \frac{2G}{3} \\ K - \frac{2G}{3} & K + \frac{4G}{3} & K - \frac{2G}{3} \\ K - \frac{2G}{3} & K - \frac{2G}{3} & K + \frac{4G}{3} \end{matrix} \right) \left( \begin{matrix} \epsilon_{xx} \\ \epsilon_{yy} \\ 0 \end{matrix} \right)$$

and can be expressed with Poisson’s number $\nu$ and elastic modulus E

$$\left( \begin{matrix} \sigma_{xx} \\ \sigma_{yy} \\ \sigma_{zz} \end{matrix} \right)=\frac{1}{E}\left( \begin{matrix} 1 & -\nu& -\nu\\ -\nu& 1 & -\nu\\ -\nu& -\nu& 1 \end{matrix} \right)\left( \begin{matrix} \epsilon_{xx} \\ \epsilon_{yy} \\ 0 \end{matrix} \right)$$

The out-of-plane stress (in z-direction) for a model domain in x-y-direction is

$$\sigma_{zz}= (K - \frac{2G}{3} )(\epsilon_{xx}+\epsilon_{yy})$$

and can be expressed using the stress components $\sigma_{xx} and \sigma_{yy}$

$$\sigma_{zz}= \frac{3K -2G}{2(3K + G)}(\sigma_{xx}+\sigma_{yy})$$

or

$$\sigma_{zz}= \nu(\sigma_{xx}+\sigma_{yy})$$

where the Poisson’s number is a function of the seismic velocities

$$\nu=\frac{V_{p}^{2}-2V_{s}^{2}}{2\left( V_{p}^{2}-V_{s}^{2} \right)}$$

The strain components are

$$\epsilon_{xx}= \frac{1}{E} \sigma_{xx} -\frac{\nu}{E} \sigma_{yy}$$

$$\epsilon_{yy}= -\frac{\nu}{E} \sigma_{xx} + \frac{1}{E} \sigma_{yy}$$

where

$$E=\frac{9KG}{3K + G}$$

$$\nu=\frac{3K -2G}{2(3K + G)}$$

The hydrostatic pressure is defined by

$$P=- \frac{1}{3}\left( \sigma_{xx} +\sigma_{yy} +\sigma_{zz} \right)$$

Hence, the deviatoric stress (2nd invariant of the deviatoric stress tensor) is given by

$$dQ=\sqrt{J_{2}}=\sqrt{\frac{1}{2}\left( \left( \sigma_{xx}+P \right)^{2}+\left( \sigma_{yy}+P \right)^{2}{+ \left( \sigma_{zz}+P \right)}^{2}+2\sigma_{xy}^{2} \right)}$$
