# Supplementary figures and images for: Modeling earthquake-induced wavefields and stresses in alpine mountains with extreme topography
Source: Sci Rep. 2025 Jul 4;15:23914. doi: 10.1038/s41598-025-08218-5 (PMC12227684; doi:10.1038/s41598-025-08218-5)

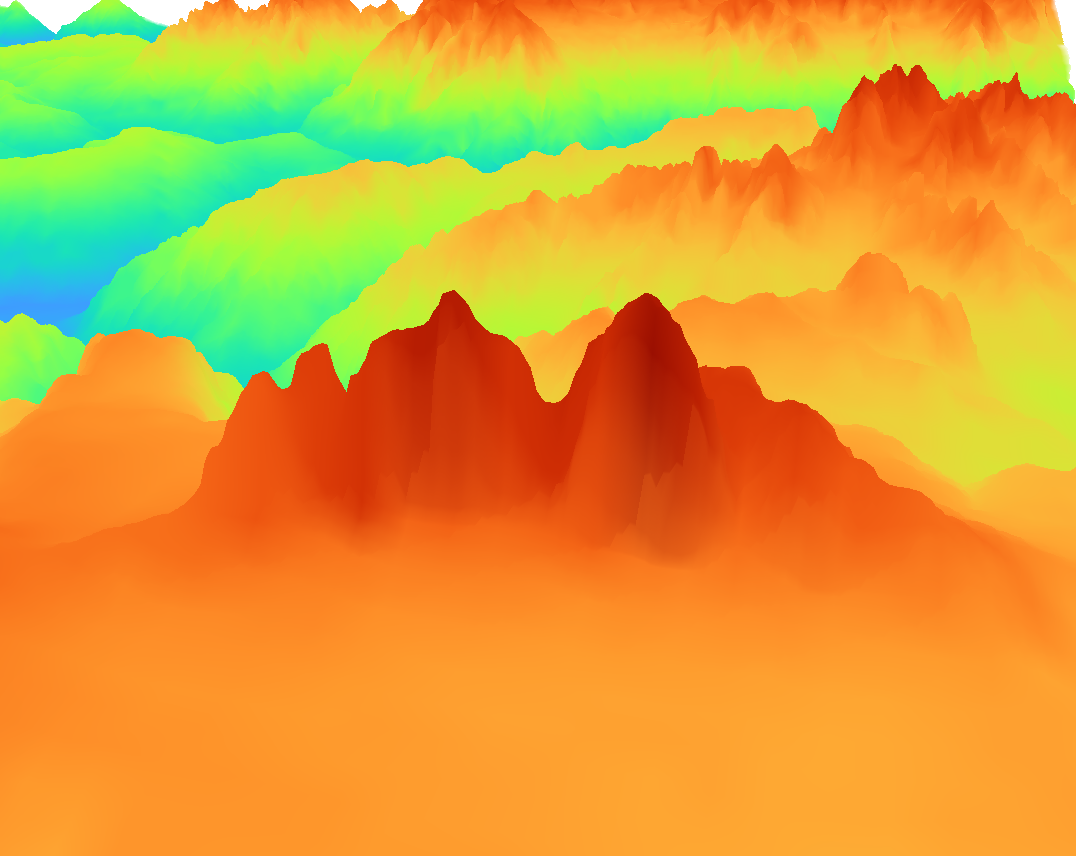

Supplement: Supplementary file 2 — Supplementary Material 2 [file 41598_2025_8218_MOESM2_ESM.png]
